# Lenvatinib Plus Pembrolizumab vs. Chemotherapy in Pretreated Patients With Advanced Endometrial Cancer: A Cost-Effectiveness Analysis

**DOI:** 10.3389/fpubh.2022.881034

**Published:** 2022-05-10

**Authors:** Mingyang Feng, Yue Chen, Yang Yang, Qiu Li

**Affiliations:** ^1^Department of Medical Oncology, Cancer Center, West China Hospital, Sichuan University, Chengdu, China; ^2^West China Biomedical Big Data Center, Sichuan University, Chengdu, China; ^3^National Medical Products Administration Key Laboratory for Clinical Research and Evaluation of Innovative Drugs, Clinical Trial Center, West China Hospital, Sichuan University, Chengdu, China

**Keywords:** cost-effectiveness, lenvatinib, pembrolizumab, endometrial cancer, immunotherapy combined therapy

## Abstract

**Background:**

In the international, randomized, open-label, phase 3 study 309-KEYNOTE-775 trial, lenvatinib plus pembrolizumab (LP) showed improved progression-free survival (PFS) and overall survival (OS) compared with chemotherapy in pretreated patients with advanced endometrial cancer. This study aimed to investigate whether LP is cost-effective compared with chemotherapy.

**Materials and Methods:**

The clinical data for this model was derived from the 309-KEYNOTE-775 trial. Costs and utilities were either derived from the standard fee database or extracted from previously published literature. A three-state Markov model was developed to simulate the disease process of patients with advanced endometrial cancer. One-way sensitivity analyses were conducted to investigate the impact of variables in the analysis model. Probabilistic sensitivity analysis was performed based on 10,000 Monte-Carlo simulations. A subgroup analysis was performed to test whether LP is cost-effective in patients with mismatch repair–proficient (pMMR) disease.

**Results:**

Lenvatinib plus pembrolizumab provided an incremental 0.64 quality-adjusted life years (QALYs) with an incremental cost of $241,278.18, compared with chemotherapy, resulting in the incremental cost-effectiveness ratio (ICER) of $378,251.44/QALY, which exceeded the willingness to pay (WTP) threshold. While in the pMMR subgroup, the ICER increased to $413,256.68/QALY. The variance of the utility of PFS state, the cost of LP, and the utility of the progressive disease state were the most influential factors in the sensitivity analysis.

**Conclusion:**

Under the current WTP threshold, LP is not cost-effective compared with chemotherapy in pretreated patients with advanced endometrial cancer.

## Introduction

The incidence and mortality of endometrial cancer are both increasing in the United States, with an estimated 65,950 new cases and 12,550 deaths in 2022 (1). Women with advanced endometrial cancer are faced with low 5-year relative survival rates of 20% and limited treatment options following initial systemic therapy (2). Pembrolizumab, a monoclonal antibody targeting programmed cell death 1(PD-1), was approved for the second-line treatment of metastatic or recurrent endometrial cancer with microsatellite instability-high (MSI-H) or mismatch repair-deficient (dMMR) status (3, 4). In the phase II trial KEYNOTE-158, pembrolizumab showed a 57% response rate in MSI-H endometrial cancers (5). However, for the more prevalent microsatellite stable (MSS) or mismatch repair–proficient (pMMR) cancers, pembrolizumab has shown less effective (6).

Lenvatinib, an oral multitargeted tyrosine kinase inhibitor of vascular endothelial growth factor receptors (VEGFR) 1-3, fibroblast growth factor receptors (FGFR) 1-4, platelet-derived growth factor receptor (PDGFR)-α, RET, and KIT (7), displayed anti-tumor activity and has been approved for several solid tumors as a single agent or in combination. While in a phase II study of lenvatinib for recurrent endometrial cancer, the overall response rate was only 14.3% (8). The combination of lenvatinib with immune checkpoint inhibitors has been evaluated in preclinical models and has shown more potent antitumor activity than either agent alone (9, 10).

The phase 3 study 309-KEYNOTE-775 (NCT03517449) showed lenvatinib plus pembrolizumab (LP) prolonged progression-free survival (PFS) and overall survival (OS), compared with chemotherapy among platinum-based chemotherapy pretreated patients with advanced endometrial cancer (11). One of the greatest challenges oncologists face today is that new therapeutic approaches are frequently associated with higher costs than previous standard techniques and, in some cases, with only marginal improvement in outcomes. This cost-effectiveness analysis aimed to compare LP vs. chemotherapy in pretreated patients with advanced endometrial cancer.

## Materials and Methods

This analysis used a mathematical modeling approach using inputs from the 309-KEYNOTE-775 trial, databases, and academic literature, following the Consolidated Health Economic Evaluation Reporting Standards (CHEERS) checklist (12).

### Patients and Interventions

The clinical data of patients with advanced endometrial cancer were derived from the multicenter, open-label, randomized phase 3 study (309-KEYNOTE-775) (11). Eligible patients had advanced, recurrent, or metastatic endometrial cancer who had disease progression after the receipt of one previous platinum-based chemotherapy regimen, and were randomized (1:1) to the LP group or chemotherapy group. In the LP group, patients received cycles of lenvatinib at a dose of 20 mg, administered orally once daily, plus pembrolizumab at a dose of 200 mg, administered intravenously every 3 weeks. In the chemotherapy group, patients received doxorubicin at a dose of 60 mg/m^2^ of body-surface area, administered intravenously every 3 weeks, and paclitaxel at a dose of 80 mg/m^2^, administered intravenously weekly (with a cycle of 3 weeks on and 1 week off). Before randomization, the treating physician would choose chemotherapy with doxorubicin or paclitaxel for each eligible patient, and MMR status was determined with biopsy specimens for each patient by pathologist evaluation. As a result, 827 patients (697 in the pMMR population and 130 in the dMMR population) were randomly assigned to a treatment group and the demographic and disease characteristics of the patients at baseline were balanced between the treatment groups (11). Treatment with one previous platinum-based therapy was reported for 79.3% of the patients in the LP group and 75.7% of those in the chemotherapy group (11).

### Model Construction

**Figure 1:**
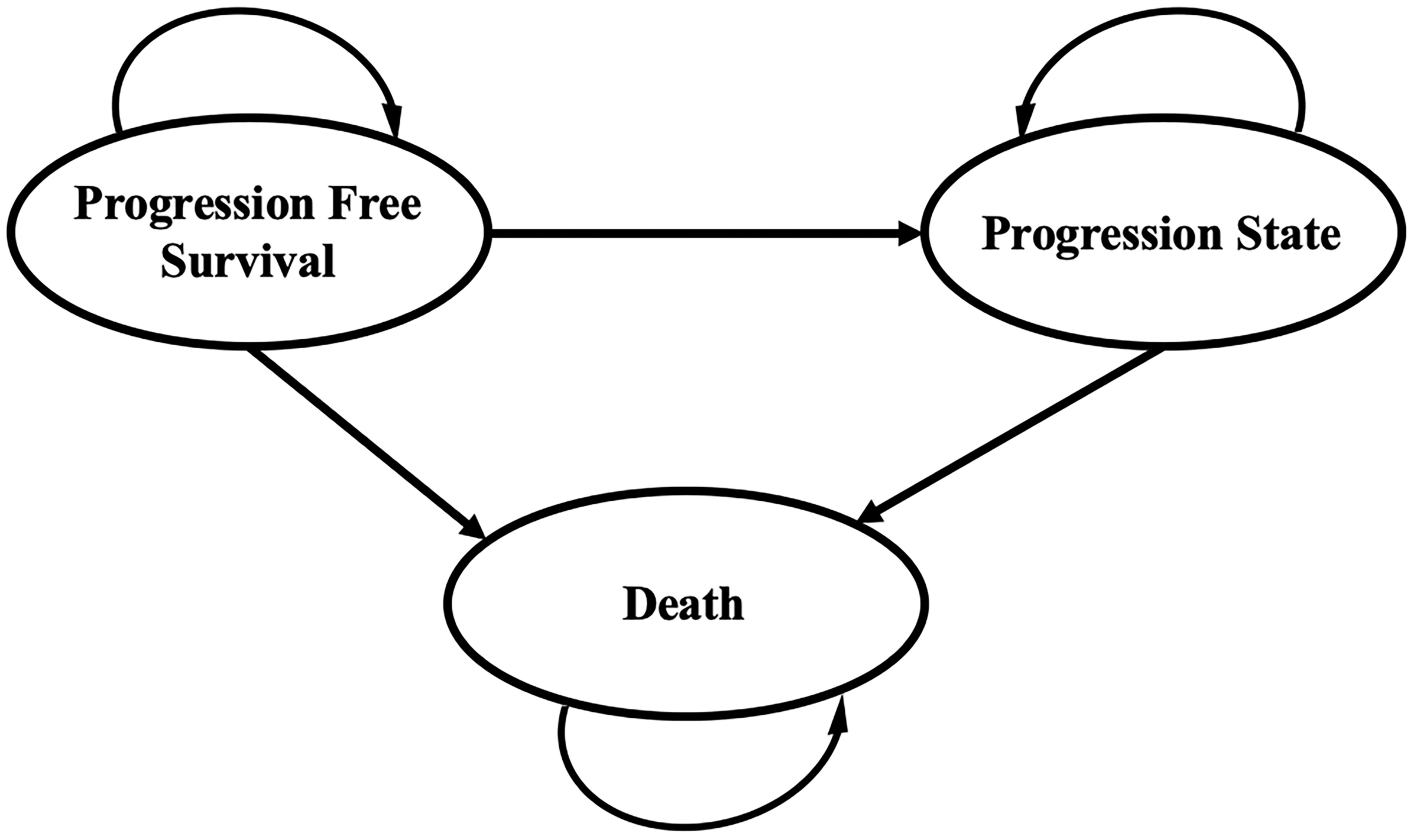
Markov model for advanced endometrial cancer. A Markov model comprising three health states was built.

A Markov decision model was developed with TreeAge Pro 2020 software (TreeAge, Williamstown, MA, USA) to simulate the disease process of patients with advanced endometrial cancer. The model structure comprised three mutually exclusive health states: PFS, progressive disease (PD), and death. All of the patients started in the PFS state and the cycle length was assumed as 1 month. Patients either stayed in the initial health status or progressed during each cycle over a lifetime horizon, as shown in Figure 1. To extrapolate the transition probabilities, the original data were extracted from the survival curves in the 309-KEYNOTE-775 trial by WebPlotDigitizer (Version:4.4; https://automeris.io/WebPlotDigitizer) (11), and these data were then used to fit parametric survival models using the algorithm derived by Hoyle et al. (13). Furthermore, we validated the results of transition probabilities using the formula: P (1 month) = 1 – (0.5) ^(1/median time to event)^, which was derived from the equations: P = 1–e^−R^ and R = –ln [0.5]/(time to event/number of treatment cycles).

### Costs

Costs were estimated from the U.S.-payer's perspective. Costs in this study were derived from the *Red Book*^®^ online database (http://www.micromedexsolutions.com, accessed on Feb 10, 2022) or previously published literature. Direct medical costs were considered, such as costs of testing for dMMR/MSI-H status, drug acquisition costs, costs of administration, disease management costs, and costs of managing adverse events (AEs) (grade ≥ 3). To more accurately calculate the costs for drug acquisition in patients with lenvatinib, we used the median dose intensity of lenvatinib of 13.8 mg/day, as specified in the 309-KEYNOTE-775 trial (11), rather than a standard dose of 20 mg/day. For patients with the progressed disease, there was no standard third-line therapy recommended by NCCN guidelines (4), and the detailed information on subsequent therapy was not specified in the 309-KEYNOTE-775 trial. Therefore, we assumed that they crossed to the other group as subsequent therapy after the failure of treatment, and the acceptance rates of subsequent therapy were assumed to be 28.0 and 48.1% in the LP group and chemotherapy group, respectively (11). Moreover, the costs of palliative care were taken into consideration as transition costs in patients who entered the death state (14). Based on the mean body surface area of 1.71 m^2^ (15), the costs for each cycle were calculated. Costs and benefits were discounted to present values at 3% each year. Half-cycle corrections were also applied. All costs were inflated to December 2021 using consumer price index (CPI) calculators (available online at: http://www.bls.gov/data/#calculators), which are listed in **Table 2**. The incremental cost-effectiveness ratio (ICER) was defined as a ratio of incremental costs to incremental benefits.

### Health Outcomes

In all populations of the 309-KEYNOTE-775 trial, the median PFS was 7.2 months in the LP group, and 3.2 months in the chemotherapy group. The median OS of the LP group and the chemotherapy group was 18.3 and 11.4 months, respectively (11). Other clinical efficacy and the proportion of patients with grade 3–4 AEs are shown in Table 1.

**Table 1:** Clinical efficacy and proportion of patients with grade 3–4 adverse events.

	**LP**	**Chemotherapy**
**Clinical efficacy-months**		
**Overall population**		
Median OS	18.3	11.4
Median PFS	7.2	3.8
**pMMR population**		
Median OS	17.4	12
Median PFS	6.6	3.8
**Proportion of patients with grade 3-4 AE**		
Hypertension	0.379	0.023
Diarrhea	0.076	0.021
Decreased appetite	0.079	0.005
Weight decrease	0.103	0.003
Anemia	0.062	0.147
Neutropenia	0.017	0.258
Neutrophil count decreased	0.017	0.212
WBC decreased	0.001	0.103

*LP, lenvatinib plus pembrolizumab; OS, overall survival; PFS, progression-free survival; pMMR, mismatch repair-proficient; AE, adverse event; WBC, white blood cell*.

Quality-adjusted life-years (QALYs) were estimated for the different treatments. QALYs were calculated as the duration in a health state multiplied by the utility weight of the corresponding health state (18). The Euro-Qol five-dimensional questionnaire (EQ-5D) encompasses a descriptive system of health-related quality of life expressed as utility indexes, ranging from perfect health (1) to death (0) (19). According to a previously published cost-effectiveness analysis, the utilities of patients with advanced endometrial cancer were identified as 0.817 for PFS, 0.779 for PD, and 0 for death (14).

### Sensitivity Analysis

Input data and ranges in the sensitivity analyses are shown in Table 2. One-way sensitivity analyses were conducted to investigate the impact of variables on the Markov model by varying variables with a range of ±25%, as shown in the tornado diagram. Probabilistic sensitivity analysis was conducted using a Monte-Carlo simulation of 10,000 patients by the simultaneous and random preset variation of parameters to evaluate optimal strategies at different hypothetical willingness-to-pay thresholds (WTP). Cost-effectiveness acceptability curves were developed to reflect the probability that treatment becomes cost-effective by varying ceiling ratios (19). A WTP threshold of $100,000 per QALY was applied.

**Table 2:** Input parameters and ranges.

	**LP**	**Chemo**	**Lower value**	**Upper value**	**Distribution**	**References**
**Costs ($, 2021 Dec)**						
**Costs for PFS per month**						
Lenvatinib	$16,166.98		$12,125.24	$20,208.73	Gamma	Red book
Pembrolizumab	$16,429.87		$12,322.40	$20,537.34	Gamma	Red book
Doxorubicin		$164.16	$123.12	$205.20	Gamma	Red book
Paclitaxel		$205.20	$153.90	$256.50	Gamma	Red book
Costs of administration per 10 mins	$55.98		$41.99	$69.98	Gamma	Thurgar et al. (14)
Costs of drug acquisition	$32,820.76	$765.31				
Hypertension	$7,965.60		$5,974.20	$9,957.00	Gamma	Arondekar et al. (16)
Diarrhea	$7,795.80		$5,846.85	$9,744.75	Gamma	Arondekar et al. (16)
Anemia	$14,314.20		$10,735.65	$17,892.75	Gamma	Le et al. (17)
Neutropenia	$14,429.73		$10,822.30	$18,037.16	Gamma	Thurgar et al. (14)
Neutrophil count decreased	$14,429.73		$10,822.30	$18,037.16	Gamma	Thurgar et al. (14)
WBC decreased	$7,071.65		$5,303.74	$8,839.56	Gamma	Thurgar et al. (14)
Costs of AE management	$3,611.44	$9,614.54				
Costs of disease management	$360.42		$270.32	$450.53	Gamma	Thurgar et al. (14)
Total	$36,792.62	$10,740.27				
**Costs for PD per month**						
Costs of disease management	$360.42		$270.32	$450.53	Gamma	Thurgar et al. (14)
Subsequent therapy	$2,906.36	$17,523.89			Gamma	This study
Total	$3,266.78	$17,884.31				
Testing for dMMR/MSI-H status-one set	$666.40		$499.80	$833.00	Gamma	Thurgar et al. (14)
Costs of palliative care-one set	$11,266.07		$8,449.55	$14,082.59	Gamma	Thurgar et al. (14)
**Transition probabilities in all population**						
PPFS-PFS	0.911	0.814			Fixed	This study
PPFS-PD	0.052	0.127			Fixed	This study
PPFS-death	0.037	0.059			Fixed	This study
PPD-PD	0.951	0.908			Fixed	This study
PPD-death	0.049	0.092			Fixed	This study
**Transition probabilities in pMMR population**						
PPFS-PFS	0.895	0.809			Fixed	This study
PPFS-PD	0.066	0.135			Fixed	This study
PPFS-death	0.039	0.056			Fixed	This study
PPD-PD	0.937	0.904			Fixed	This study
PPD-death	0.063	0.096			Fixed	This study
**Utilities**						
PFS state	0.817		0.613	1.000	Beta	Thurgar et al. (14)
PD state	0.779		0.584	0.974	Beta	Thurgar et al. (14)
Death	0.000				Fixed	

*LP, lenvatinib plus pembrolizumab; PFS, progression-free survival; WBC, white blood cell; AE, adverse event; PD, progressive disease; MSI-H, microsatellite instability–high; dMMR, mismatch repair–deficient; P, transition probability; pMMR, mismatch repair-proficient*.

### Subgroup Analysis

A subgroup cost-effectiveness analysis was performed for the pMMR population, and the transition probabilities were extrapolated using the survival data supplied by the 309-KEYNOTE-775 trial. In the pMMR subgroup (*n* = 697, 84.3%), the median PFS was 6.6 months in the LP group, 3.8 months in the chemotherapy group, and the median OS was 17.4 and 12.0 months in the LP group and the chemotherapy group, respectively (11).

## Results

### Costs Outcomes

In terms of PFS state costs, the costs of drug acquisition accounted for most in the LP group ($32,820.76 per month), while the costs of grade 3–4 AEs management accounted for the majority of the chemotherapy group ($9,614.54 per month), as shown in Table 2. The disease management costs were assumed to be the same in the two groups at $360.42 per month. As for the costs for PD state, the total cost was $3,266.78 per month for the LP group and $17,884.31 per month for the chemotherapy group, including costs of disease management and subsequent therapy. Overall, the cumulative costs were $432,785.93 for the LP group, which was higher than that of $191,507.75 for the chemotherapy group (as shown in Table 3).

**Table 3:** Results of the cost-effectiveness analysis in all populations.

	**LP**	**Chemo**
**Base-case analysis**		
**Costs ($)**		
Costs for PFS state	390,064.27	55,989.90
Costs for PD state	42,721.66	135,517.85
Total costs	432,785.93	191,507.75
Incremental costs	241,278.18	
**Effectiveness (QALYs)**		
Effectiveness for PFS state	0.71	0.33
Effectiveness for PD state	0.72	0.46
Total effectiveness	1.43	0.79
Incremental effectiveness	0.64	
**Cost/Effectiveness**	302,626.35	241,734.90
**ICER ($ per QALY)**	378,251.44	
**Monte-Carlo simulation (10000x)**		
Mean costs ($)	430,736.70	192,160.92
SD	96,209.99	34,481.06
Mean effectiveness (QALYs)	1.43	0.79
SD	0.25	0.14

*LP, lenvatinib plus pembrolizumab; PFS, progression-free survival; PD, progressive disease; QALY, quality-adjusted life-year; ICER, incremental cost-effectiveness ratio; SD, standard deviation*.

### Health Outcomes

Based on the aforementioned algorithm, calibrated transition probabilities are listed in Table 2, and the curves of the fitted parametric model are displayed in Supplementary Materials (Supplementary Figures S1, S2). The overall effectiveness in the LP group was higher than that in the chemotherapy (1.43 vs. 0.79 QALYs), as shown in Table 3.

### Base-Case Results

The base-case results of the analysis are presented in Table 3. Treatment with LP was estimated to generate an incremental 0.64 QALYs with incremental costs of $241,278.18 compared with the chemotherapy group, resulting in an ICER of $378,251.44/QALY.

### Sensitivity Analysis

**Figure 2:**
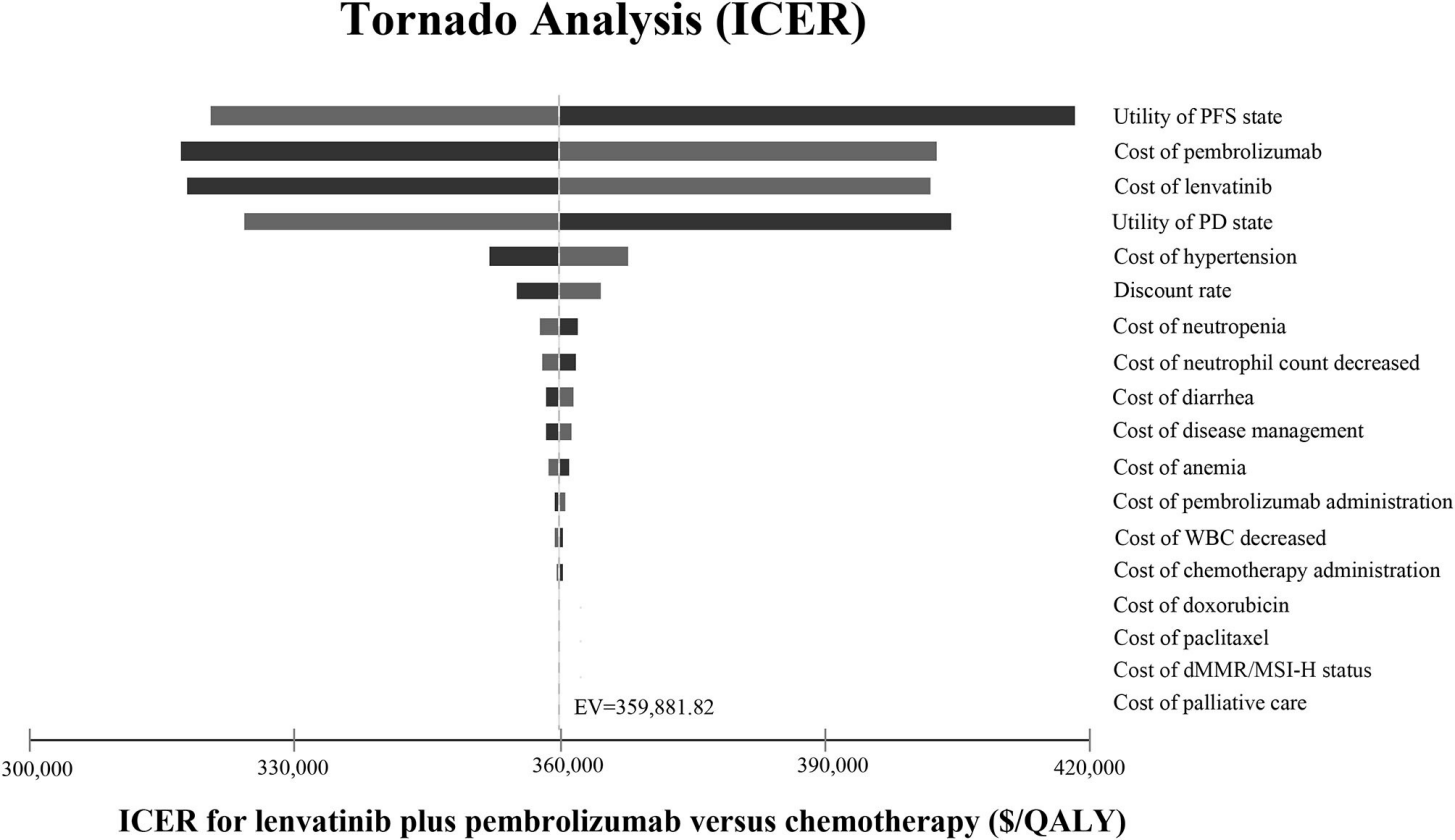
Tornado diagram. The tornado diagram shows the one-way sensitivity analyses within the appropriate range for each variable. ICER, incremental cost-effectiveness ratio; PFS, progression-free survival; PD, progressive disease; WBC, white blood cell; dMMR, mismatch repair–deficient; MSI-H, microsatellite instability–high; QALY, quality-adjusted life year; EV, expected value.

**Figure 3:**
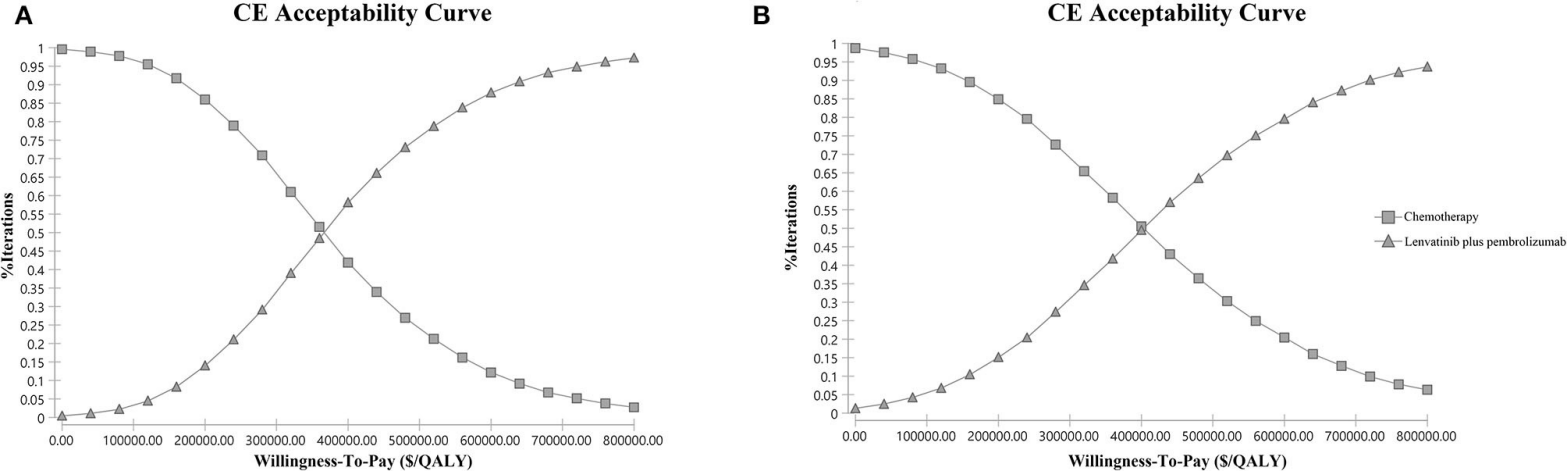
Probabilistic sensitivity analysis. **(A)** Overall study population. **(B)** the pMMR subgroup. The cost-effectiveness acceptability curve indicates the probability (*y*-axis) of lenvatinib plus pembrolizumab being cost-effective compared with chemotherapy given the threshold value (*x*-axis). CE, cost-effectiveness; QALY, quality-adjusted life year.

The one-way sensitivity analyses are displayed in the tornado diagram in Figure 2, where the variables were changed across a range of ±25%. The utility of PFS state, the cost of lenvatinib and pembrolizumab, and the utility of PD state were the most influential factors in this study. The result of the Monte-Carlo simulation of 10,000 patients showed that the mean cost and effectiveness gained were $430,736.70 ± 96,209.99 and 1.43 ± 0.25 QALYs for the LP group, while $192,160.92 ± 34,481.06 and 0.79 ± 0.14 QALY for the chemotherapy group. The probabilistic sensitivity analysis indicated that the LP was not likely to be accepted until the WTP rose above $360,000 (Figure 3A).

### Subgroup Analysis

In the subgroup analysis of the pMMR population, the cumulative costs and effectiveness were $367,346.00 and 1.21 QALYs for the LP group, and $189,197.40 and 0.78 QALYs for the chemotherapy group, resulting in the ICER at $413,256.68/QALY, as shown in Table 4. The probabilistic sensitivity analysis indicated that LP was more likely to be accepted when the WTP rose over $400,000 (Figure 3B).

**Table 4:** Results of the cost-effectiveness analysis in patients with pMMR.

	**LP**	**Chemo**
**Base-case analysis**		
**Costs ($)**		
Costs for PFS state	329,567.32	54,243.43
Costs for PD state	37,778.68	134,954.02
Total costs	367,346.00	189,197.40
Incremental costs	178,148.60	
**Effectiveness (QALYs)**		
Effectiveness for PFS state	0.60	0.32
Effectiveness for PD state	0.61	0.46
Total effectiveness	1.21	0.78
Incremental effectiveness	0.43	
**Cost/Effectiveness**	303,358.70	242,609.04
**ICER ($ per QALY)**	413,256.68	
**Monte-Carlo simulation (10000x)**		
Mean costs ($)	366,289.42	189,091.23
SD	80,918.20	34,048.63
Mean effectiveness (QALYs)	1.21	0.78
SD	0.21	0.14

*LP, lenvatinib plus pembrolizumab; PFS, progression-free survival; PD, progressive disease; QALY, quality-adjusted life-year; ICER, incremental cost-effectiveness ratio; SD, standard deviation*.

## Discussion

Based on the phase 3 trial 309-KEYNOTE-775, our study indicated LP cost more ($432,785.93 vs. $191,507.75) and yielded more health outcomes than chemotherapy (1.43 vs. 0.79 QALYs), resulting in the ICER of $378,251.44/QALY, which was much beyond the prespecified WTP threshold ($100,000/QALY), suggesting that LP is not a cost-effective choice compared with chemotherapy. One-way sensitivity analysis and probabilistic sensitivity analysis both suggested that this result was robust. For patients with pMMR disease, the combination strategy had shown less cost-effective than chemotherapy, as the ICER increased to $413,256.68/QALY.

The most influential factors driving our models were the utility of the PFS state, the cost of lenvatinib and pembrolizumab, and the utility of the PD state. The ICER still exceeded the WTP threshold no matter how much the value of these variances changed. As the costs of lenvatinib and pembrolizumab both exerted a significant effect on the sensitivity analysis, decreasing the price of lenvatinib and pembrolizumab could be an efficient strategy to reduce ICER.

Pembrolizumab monotherapy may be less effective in patients with recurrent MSS/pMMR tumors (6), and the strategy of lenvatinib in combination with pembrolizumab was confirmed effective in patients with advanced endometrial cancer (20). In this cost-effectiveness analysis, we found that the ICER of combination therapy continued to increase in the pMMR subgroup, compared with the chemotherapy group. We suspected that it was due to the significantly reduced effectiveness of the LP group in the patients with pMMR disease, compared with that in the overall population (1.21 vs. 1.43 QALYs), as the median PFS and median OS both reduced in the LP group in patients with pMMR disease (Table 1).

To our knowledge, there has been a series of cost-effective analyses evaluating different kinds of treatment in advanced endometrial cancer. The cost-effectiveness of pembrolizumab monotherapy was evaluated in pretreated advanced endometrial cancer, and positive results were obtained that pembrolizumab monotherapy was a highly cost-effective treatment option when compared with chemotherapy, especially in patients with dMMR/MSI-high disease (14, 21). However, when pembrolizumab is combined with lenvatinib, the conclusions would be changed. For instance, LP has been evaluated compared with carboplatin/paclitaxel in the first-line therapy for patients with advanced endometrial cancer (22). In this study, the authors found that LP was dominated by carboplatin/paclitaxel in patients with MSS disease, and was not cost-effective in the MSI-high model. Moreover, the cost-effectiveness of LP in patients with recurrent pretreated MSS endometrial cancer was evaluated by Barrington et al. (23), and consistent with our conclusion, they found that LP was not cost-effective. Notably, their analysis was conducted based on the results of phased 2 trial (20), and our results would make a powerful addition to this topic. All of these studies warn us that performing reasonable economic evaluation has become an important and indispensable part of the cancer-treat resources allocation process, and could guide clinical practice.

The limitations of our study are as follows. First, the 309-KEYNOTE-775 trial collected the quality of life-related information with the health-related quality of life instrument QLQ-C30. The utility of disease pattern in our study was otherwise extracted from previously published advanced endometrial cancer economic model, in which utilities were calculated from EQ-5D data in a clinical trial, which may not accurately reflect the patients' quality of life in the 309-KEYNOTE-775 trial. Second, as there was no standard third-line therapy after the failure of the second-line treatment of recurrent or metastatic endometrial carcinoma according to NCCN guidelines (4), we assumed the patients of the two groups switched to the other. The assumption may not be very accurate, but we believe it could somewhat reflect the real situation. The acceptance rates of subsequent therapy were extracted from the 309-KEYNOTE-775 trial (11). Another is that, in our analysis, we only considered the costs of AEs management, and ignored the disutility caused by AEs, as there remained controversial opinions on the values of disutility in different AEs and diseases. While in our sensitivity analysis, we found that the utility of the PFS state impacts most of the model outcomes, but the ICER still exceeded the WTP threshold while the value of utility changed (0.613–1.000), which could confirm the robustness of our results. Moreover, costs and WTP threshold could vary between different medical centers or different countries, and this may affect the generalizability. Our sensitivity analysis was conducted by varying variables with a range of ±25%, demonstrating that the results remained robust while variables changed, and a future perspective cost-effectiveness study is expected to further verify our results.

In conclusion, our study evaluated the cost-effectiveness of LP versus chemotherapy in pretreated patients with advanced endometrial cancer and found that LP is not a cost-effective choice from a U.S.-payers' perspective, which could be considered in the decision-making process to make recommendations regarding the therapy for patients with advanced endometrial cancer. Reducing the price of LP or offering appropriate drug assistance policies might be considerable options to optimize the cost-effectiveness of LP.

## Data Availability Statement

The original contributions presented in the study are included in the article/Supplementary Material, further inquiries can be directed to the corresponding author.

## Author Contributions

Material preparation, data collection, and analysis were performed by MF and YC. The first draft of the manuscript was written by MF. The revised manuscript was written by MF, YC, and YY. All authors contributed to the study conception and design, and read and approved the final manuscript.

## Funding

This work was supported by the 1.3.5 Project for Disciplines of Excellence, West China Hospital, Sichuan University (Grant Nos. ZYJC18008 and ZYJC18010). The funding source was not involved in the study design, data collection, data analysis, data interpretation, the writing of this article, or the decision to submit the article for publication.

## Conflict of Interest

The authors declare that the research was conducted in the absence of any commercial or financial relationships that could be construed as a potential conflict of interest.

## Publisher's Note

All claims expressed in this article are solely those of the authors and do not necessarily represent those of their affiliated organizations, or those of the publisher, the editors and the reviewers. Any product that may be evaluated in this article, or claim that may be made by its manufacturer, is not guaranteed or endorsed by the publisher.
