# Supplementary material for: Lenvatinib Plus Pembrolizumab vs. Chemotherapy in Pretreated Patients With Advanced Endometrial Cancer: A Cost-Effectiveness Analysis
Source: Front Public Health. 2022 May 10;10:881034. doi: 10.3389/fpubh.2022.881034 (PMC9127138; doi:10.3389/fpubh.2022.881034)
Supplement: Supplementary file 1 [file Presentation_1.PPTX]

## Slide 1
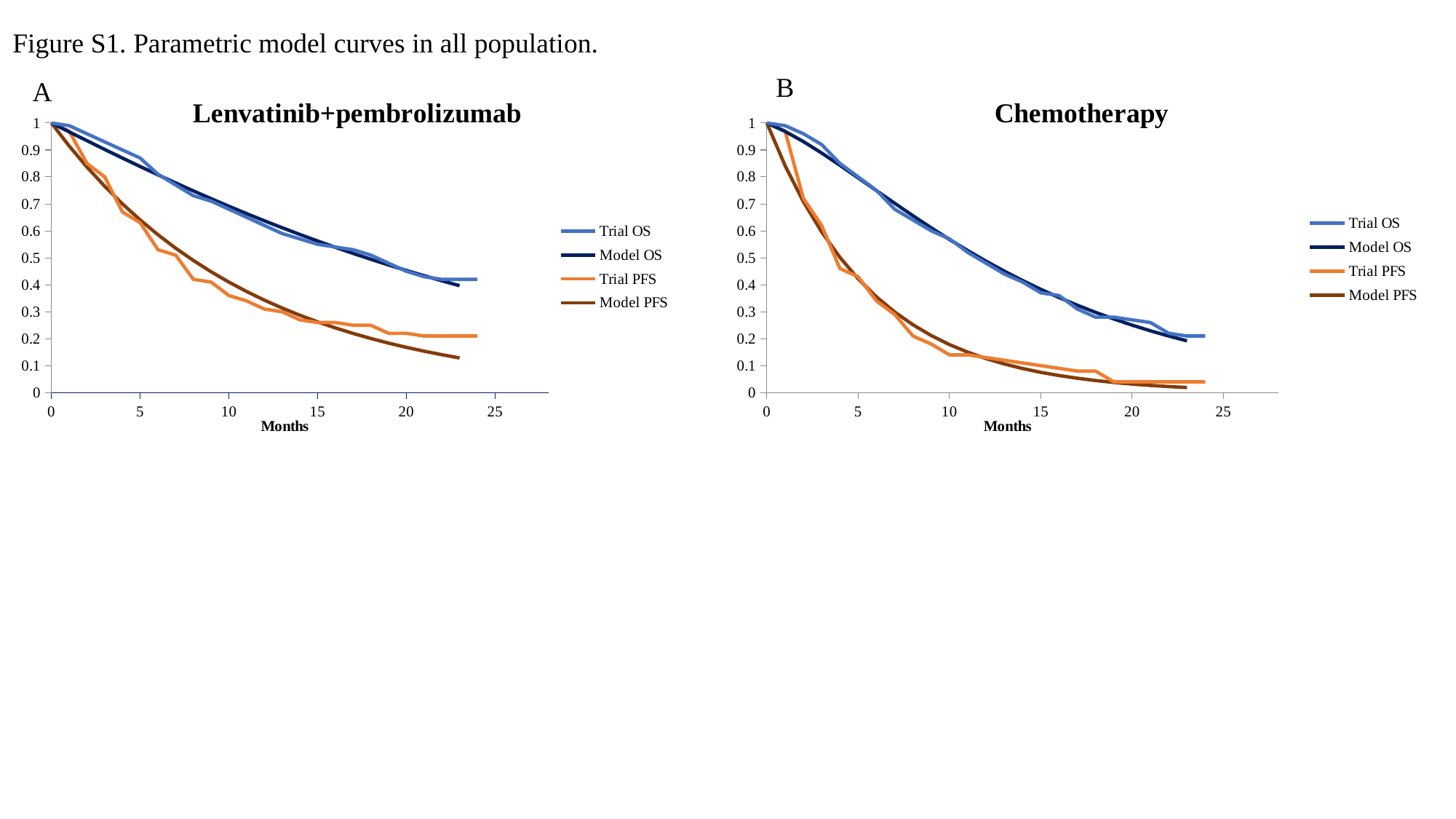

Figure S1. Parametric model curves in all population.
B
A
### Chart: Lenvatinib+pembrolizumab
| Category | | | | |
|---|---|---|---|---|
### Chart: Chemotherapy
| Category | | | | |
|---|---|---|---|---|

## Slide 2
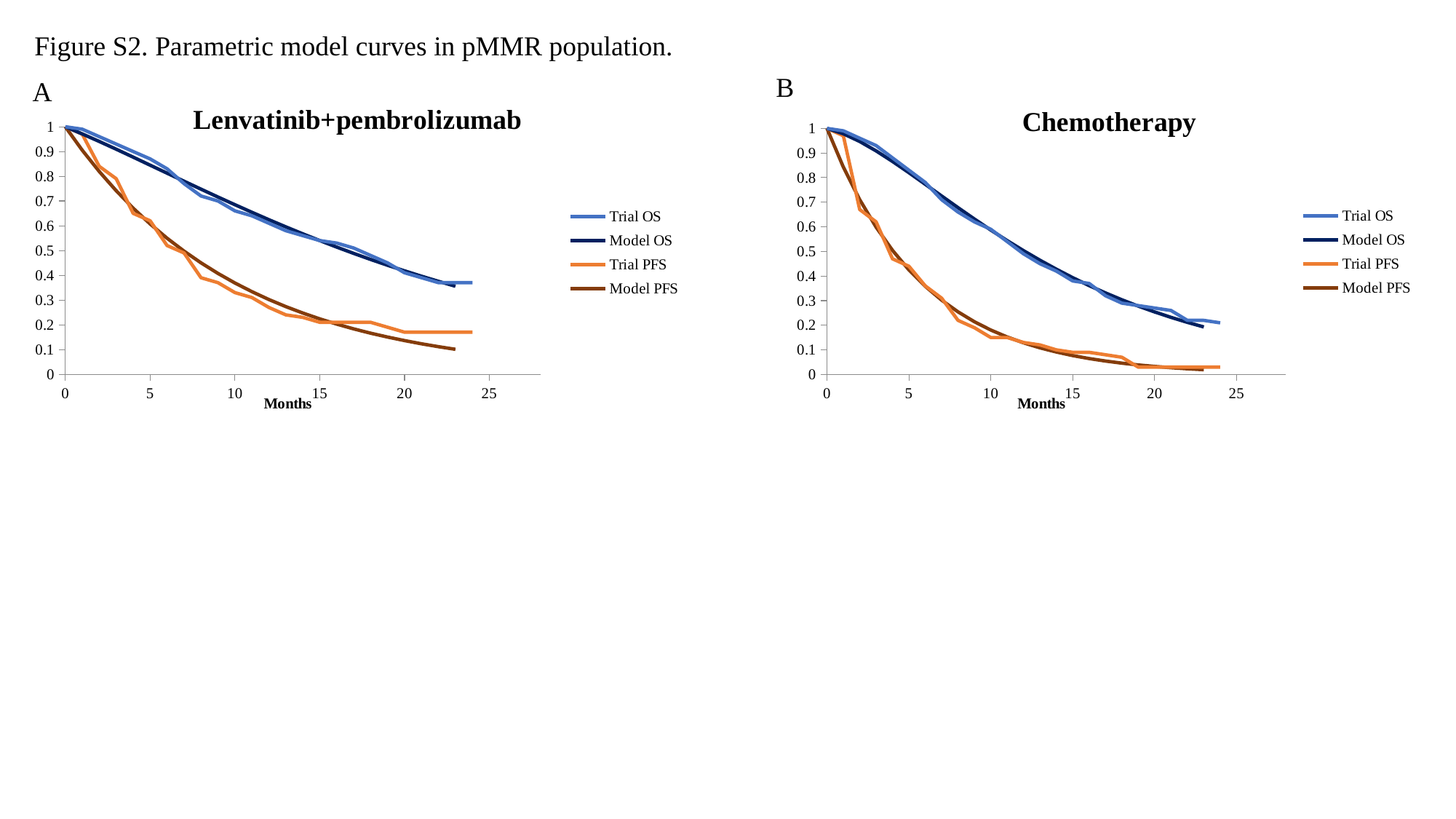

Figure S2. Parametric model curves in pMMR population.
B
A
### Chart: Lenvatinib+pembrolizumab
| Category | | | | |
|---|---|---|---|---|
### Chart: Chemotherapy
| Category | | | | |
|---|---|---|---|---|
